# Bilateral and multiple sub-internal limiting membrane hemorrhages in a familial retinal arteriolar tortuosity patient by Valsalva-like mechanism: an observational case report

**DOI:** 10.1186/s12886-020-01413-0

**Published:** 2020-04-15

**Authors:** Ting Chen, Hongmei Zheng, Yunyun Wang, Junyi Hu, Changzheng Chen

**Affiliations:** Department of Ophthalmology, Renmin Hospital of Wuhan University, No. 9 ZhangZhiDong Street, Wuchang District, Wuhan, 430060 Hubei China

**Keywords:** Familial retinal arteriolar tortuosity, Valsalva, Neodymium yttrium aluminum garnet, Observation

## Abstract

**Background:**

Bilateral and multiple Valsalva-related sub-internal limiting membrane (ILM) hemorrhages in a familial retinal arteriolar tortuosity (FRAT) patient is rare, and we treated this patient by both observation and Neodymium yttrium aluminum garnet (Nd: YAG) laser membranotomy methods.

**Case presentation:**

A 13-year-old female student presented with sudden visual loss and central scotoma in both eyes after running 800 m at the school gym. The examination revealed six sub-ILM hemorrhages with the biggest hemorrhage measuring approximately 1.5-disc diameters (DD) in the right eye and two sub-ILM hemorrhages with the biggest one measuring 5.5 DD in the left eye. The patient was diagnosed as having Valsalva retinopathy associated with FRAT. Nd: YAG laser membranotomy was performed at the biggest hemorrhages and the rest hemorrhages were treated with observation in both eyes. The visual acuity recovered to 20/16 in the right eye and 20/20 in the left eye. Epiretinal membrane (ERM) formation was observed in the left eye.

**Conclusions:**

Nd: YAG laser could be considered for treating premacular hemorrhage in FRAT patient especially when a quick vision recovery was needed. This is the first reported case of a FRAT patient suffering from bilateral and multiple Valsalva-related sub-ILM hemorrhages which were treated by both observation and Nd: YAG laser treatment.

## Background

Familial retinal arteriolar tortuosity (FRAT) is a disorder characterized by pronounced tortuosity of the second-order and third-order retinal arterioles in the macular and peripapillary areas while sparing of the first-order arteriole and the whole venous system. The observation of male to male inheritance as well as occurrence in parents and their children in the absence of consanguinity suggest an autosomal dominant inheritance in FRAT [1]. FRAT patients are often asymptomatic but some may experience transient vision loss due to intra- or preretinal hemorrhages following physical exertion or minor trauma [1, 2]. The long-time visual prognosis is usually excellent.

The Valsalva maneuver is performed by forceful exhalation against a closed glottis causing an increase in intraabdominal and intrathoracic pressure during activities such as coughing, vomiting, straining, or weightlifting. When the increased pressure transmitted to the eye, the spontaneous rupture of superficial perifoveal retinal capillaries may happen. It can be bilateral but the unilateral characteristic is more common [3]. The hemorrhages in most cases resolve spontaneously over several weeks.

Here, we presented a case of a FRAT patient suffering from Valsalva-related retinal hemorrhages was treated with Neodymium yttrium aluminum garnet (Nd: YAG) laser membranotomy and observation methods. This case was unusual as it underwent multiple sub-internal limiting membrane (ILM) hemorrhages in both eyes in FRAT patient by a Valsalva-like mechanism, as well as both observation and Nd: YAG laser treatment were chosen in this FRAT patient.

## Case presentation

**Fig. 1:**
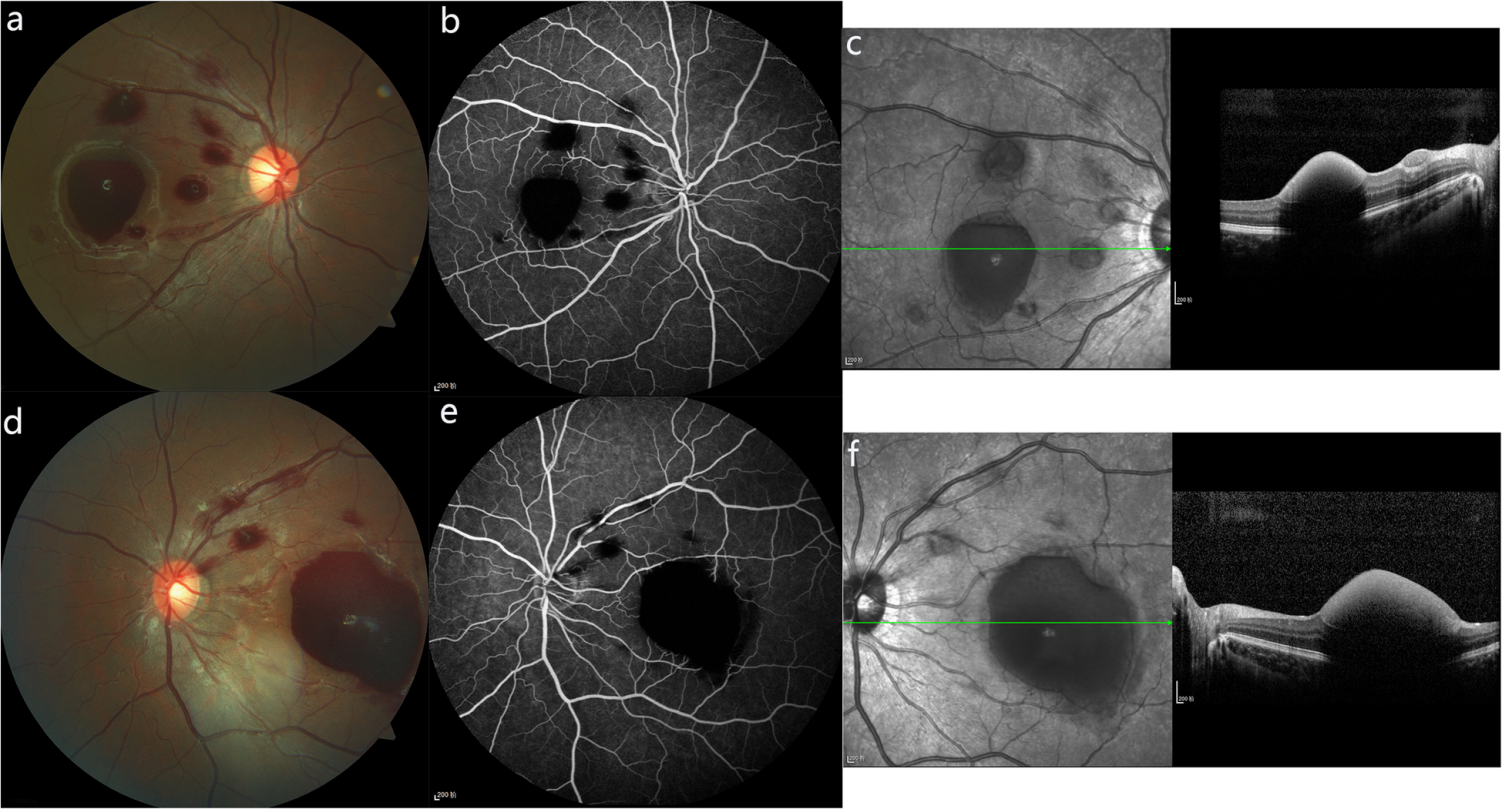
Fundus photographs, FFA and SD-OCT results of a student in both eyes. Fundus photograph showed multiple retinal hemorrhages, as well as second- and third-order retinal arteriolar tortuosity in the macula and peripapillary area in the right eye (**a**) and the left eye (**d**). FFA of the right (**b**) and the left (**e**) eye revealed retinal arteriolar tortuosity with the blocked fluorescence from the retinal hemorrhages. SD-OCT demonstrated a dome-shaped lesion with a hyper-reflective band and a hypo-reflective area beneath consistent with blood in the right (**c**) and the left eye (**f**)

**Fig. 2:**
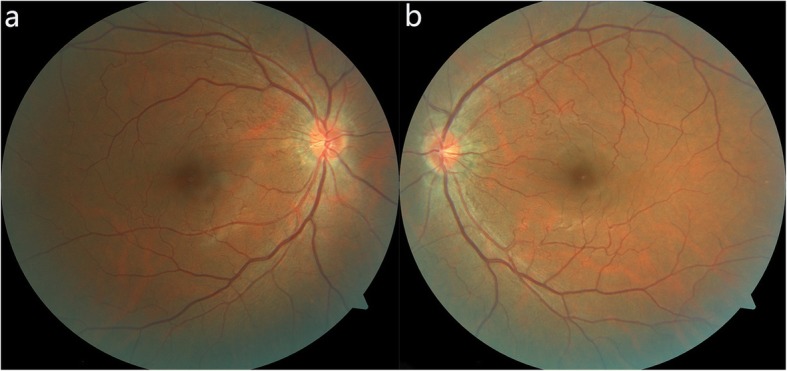
Fundus photographs of this familial retinal arteriolar tortuosity (FRAT) patient’s mother

A 13-year-old female student was admitted to our department because of sudden visual loss and central scotoma in both eyes after running 800 m at the school gym for 1 week. She had no history of ocular, systemic disease or trauma. The presenting best corrected visual acuity (BCVA) was 20/100 in the right eye and 20/160 in the left eye. Her intraocular pressures were 20 mmHg in the right eye and 18 mmHg in the left eye. Her pupillary response, ocular motility, external and anterior segment examinations were unremarkable. Dilated fundus examination revealed six retinal hemorrhages with the biggest hemorrhage measuring approximately 1.5-disc diameters (DD) in the right eye and two retinal hemorrhages with the biggest one measuring 5.5 DD in the left eye. The hemorrhages showed a double-ring sign, with the outer ring representing the subhyaloid bleed and the inner ring representing the sub-ILM hemorrhage. Several areas of intraretinal hemorrhages located in and around the posterior pole were also observed. Moreover, fundus examination of both eyes showed the tortuosity of second and higher order arterioles which were consistent with FRAT (Fig. 1). Her mother has similar arteriolar tortuosity in the fundus (Fig. 2). Spectral-domain optical coherence tomography (SD-OCT; Spectralis HRA + OCT; Heidelberg Engineering, Heidelberg, Germany) showed a dome-shaped lesion with a hyper-reflective band and a hypo-reflective area beneath consistent with blood at the macula in both eyes. From the OCT, the hemorrhages were confined below the ILM (Fig. 1). Findings from fundus fluorescein angiography (FFA) in both eyes showed the blocked fluorescence from the retinal hemorrhages, and the second- and third-order retinal arteriolar tortuosity with corkscrew shape in the posterior pole, without vascular filling defects or hyperfluorescent lesions. No lesions were detected in the venous vasculature and first-order arteries (Fig. 1). The blood pressure, biochemical test, blood cell count and coagulation were normal. Based on the clinical presentation and her history, the ultimately diagnosis of this patient was Valsalva retinopathy associated with FRAT.

**Fig. 3:**
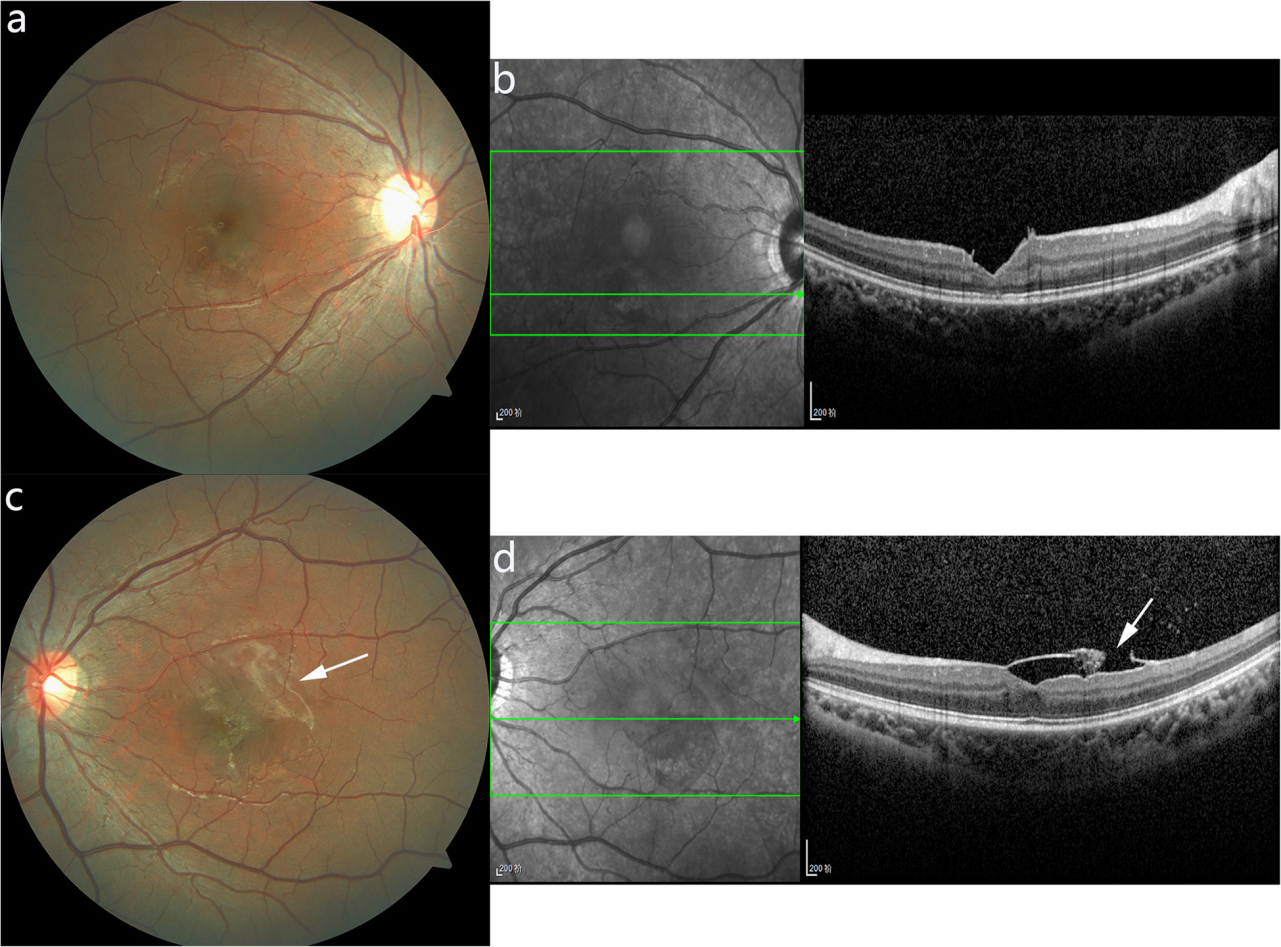
Fundus photographs and SD-OCT images 7 weeks after Nd: YAG laser membranotomy. Fundus photos showed that all retinal hemorrhages were resolved in the right (**a**) and left (**c**) eye, with the internal limiting membrane (ILM) wrinkling in the left eye (**c,** arrow). SD-OCT revealed subtle loss of foveal contour in the right (**b**) and left (**d**) eye, as well as a laser perforation in the ILM and a sub-ILM hypo-reflective area in the left eye (**d**, arrow)

As a student, she and her family request a quick vision recovery. Comparing the potential risks, benefits and costs between surgery and laser, Nd:YAG laser was chosen. Nd:YAG laser (Ultra Q Reflex, Ellex, Australia) with a Goldmann three-mirror contact lens was performed at the lower part of the maximum premacular hemorrhages in both eyes at the same time. The energy was 5–6 mJ with single pulse. Consequently, the hemorrhages were rapidly trapped into the vitreous cavity. Five days after the membranotomy, the premacular hemorrhages had drained mostly with mild vitreous hemorrhages inferiorly, and her visual acuity was improved to 20/16 in the right eye and 20/20 in the left eye. At 7 weeks, the visual acuity of both eyes were stable. All retinal hemorrhages were resolved completely with little grayish vitreous hemorrhages in both eyes and the ILM was wrinkled in the left eye (Fig. 3). OCT showed subtle loss of foveal contour in both eyes and in the left eye, a laser perforation in the ILM and hypo-reflective premacular cavity was observed (Fig. 3).

## Discussion and conclusions

FRAT is a rare disease (OMIM %180,000), with the exact etiology is unknown yet. Diagnosis of FRAT is based on the clinical feature of the second and third order tortuosity of arterioles in the peripapillary and macular areas. Moreover, when there is a family history or a positive fundus result of one family member, the diagnosis is more definite. FRAT is usually asymptomatic and the visual symptoms occur with the retinal hemorrhage. However, the hemorrhage is not associated with the severity of the arterioles tortuosity in FRAT [1]. The hemorrhages are often correlated with mild trauma, physical exertion, or other activities that could result in elevated central venous pressure (Valsalva effect) [4].

This case is unique as our FRAT patients suffering from bilateral and multiple sub-ILM hemorrhages likely because of a Valsalva effect related to the running activity. It was unclear how FART predisposes to Valsalva retinopathy. Some researchers indicated that the tortuosity of the retinal arterioles may be the result of disruption of the extracellular matrix of the arterial wall [5, 6]. Meanwhile, FRAT is thought to be an autosomal dominant disorder associated with a missense mutation in the Collagen types IV alpha-1 (*COL4A1*) gene in some cases [7]. COL4A1 is the most abundant and ubiquitous basement membrane protein and is present in the basal lamina of various ocular structures and vascular basement membranes [8, 9]. *COL4A1* mutation can affect the collagen secretion and accumulate misfolded proteins [7]. So we hypothesized that when a Valsalva-like mechanism induced an increased venous pressure, the hemorrhage originating from the fragility of the tortuous arterioles in FRAT patients happened.

The visual prognosis is often excellent either in FRAT or in Valsalva retinopathy. Following the absorption of the hemorrhages, the visual acuity usually returns to normal [10]. The primary treatment of FRAT and Valsalva retinopathy are both conservation. However, in some observed cases, irreversible visual impairment may occur because of macular pigmentary changes, epiretinal membrane (ERM) formation, or the toxicity of hemoglobin and iron on retina [11–13]. Other treatment options such as Nd: YAG laser membranotomy [14], vitrectomy [15], intravitreal injection of ranibizumab [16], preumatic displacement of the hemorrhage by gas and tissue plasminogen activator [17] and argon green laser [18] were recommended in Valsalva retinopathy, while not reported in FRAT patient until now. The other particularity of our case was that this FRAT patient was under observation for the little sub-ILM hemorrhages as well as Nd: YAG for the maximum premacular sub-ILM hemorrhages. Both therapies were effective for our patient. To our best knowledge, this was the first case report of sub-ILM hemorrhages in FRAT patient treated by Nd: YAG laser membranotomy. Although it has been advocated that if the premacular hemorrhage is < 3 DD, Nd-YAG laser should not be performed [19]. However, other study also suggested that if quicker resolution of symptoms was demanded, Nd: YAG laser is an option for premacular hemorrhage [14]. Chang et al. [20] also advised that if Nd: YAG laser is considered, it should be performed as early as possible. As it is hard to achieve the complete drainage of the clotted blood, despite successful laser perforation. As a student, our patient and her family demanded a quick resolution, so we also gave a Nd:YAG laser treatment for the right eye with the premacular hemorrhage was only 1.5 DD.

Some research showed that the ERM formation and ILM wrinkling may occur as a late complication of Nd: YAG laser therapy [21, 22]. In this case, we also observed the ERM formation in left eye. Although we reported that ERM formation occurred in one eye, it did not indicate that the complication rate of Nd: YAG laser membranotomy for treating premacular hemorrhage in FRAT patient was 50% when the studied sample was only one patient. The presence of persistent hemorrhage beneath the ILM may be the stimulus for ERM formation rather than the Nd: YAG laser treatment itself [22]. ILM’s molecular constituents include members of the laminin, nidogen, collagen IV, and proteoglycan families [23]. The mutation in the COL4A1 gene, which encodes type IV collagen in basement membranes, has been reported in FRAT [7]. Thus, it was possible that the FRAT patient may have a defect ILM due to the genetic abnormality, and it may be related to the unsealed and un-reattached ILM membrane observed in this patient. In addition, the ERM formation may originate from the persistent unsealed and un-reattached ILM membrane [22]. This opinion was verified by our patient by the evidence that the ERM formation only occurred in the left eye with unsealed ILM membrane while did not observe in the right eye. And there was investigation showed that any elevations of the ILM may seal and reattach among 2–6 months [24]. According to this view, our patient may have a structure alteration in macular in the left eye observed by OCT. Meanwhile, at the follow-up of 7 weeks, our FRAT patient did not complain of metamorphopsia. We will observe this patient for a long time.

We conducted the Nd:YAG laser membranectomy at the lower part of the maximum premacular hemorrhages. But in our patient, the location of ILM defect observed in the fundus after 7 weeks after the membranotomy was far from the inferior border of the initial sub-ILM hemorrhage, which may indicate that the laser performed at an inappropriate location. The explanation for the location of ILM defect far from the inferior border was due to the tangential traction of the ILM [25].

In conclusion, we reported a rare case of a FRAT patient suffering from bilateral and multiple Valsalva-related sub-ILM hemorrhages which were treated by both observation and Nd: YAG laser treatment. Nd: YAG laser could be considered for treating premacular hemorrhage in FRAT patient especially when a quick vision recovery was needed. We recommend that this student avoid physically intensive performances or Valsalva. The long-time efficacy and safety of Nd: YAG laser in FRAT patient needs further study.

## Data Availability

All data and materials supporting our findings are contained within this manuscript.

## Abbreviations

ILM: internal limiting membrane
FRAT: Familial retinal arteriolar tortuosity
Nd: YAG: Neodymium yttrium aluminum garnet
DD: Disc diameters
ERM: Epiretinal membrane
BCVA: Best corrected visual acuity
FFA: Fundus fluorescein angiography
*COL4A1*: Collagen types IV alpha-1
